# Correlation between hematological profile and theileriosis in Bali cattle from Muara Bulian, Jambi, Indonesia

**DOI:** 10.14202/vetworld.2019.1358-1361

**Published:** 2019-09

**Authors:** N. Aziz, M. Maksudi, Y. A. Prakoso

**Affiliations:** 1 Department of Animal Husbandry, Faculty of Animal Husbandry, University of Jambi, Muara Bulian, Jambi, 36122, Indonesia; 2 Faculty of Veterinary Medicine, University of Wijaya Kusuma Surabaya, Surabaya, East Java, 60225, Indonesia

**Keywords:** Bali cattle, CD4+/CD8+ ratio, correlation, hematological profile, prevalence, theileriosis

## Abstract

**Background and Aim:**

Theileriosis is a parasitic disease caused by the hemoprotozoan *Theileria* spp. The main transmission route of this disease is through tick vector bite. Theileriosis causes economic losses in livestock such as Bali cattle. This study aims to analyze the prevalence of theileriosis and its correlation with the hematological profile of Bali cattle from the subdistrict of Muara Bulian, Batang Hari, Jambi, Indonesia.

**Materials and Methods:**

Ninety-four blood samples were collected through jugular vein of Bali cattle. The presence of *Theileria* spp. was determined using blood smear. Routine blood tests and double-staining immunohistochemistry against CD4+ and CD8+ lymphocytes were conducted on all blood samples.

**Results:**

A total of 34.04% of the samples were infected by *Theileria* spp. Theileriosis affected only hemoglobin level (p<0.05); it did not affect the other parameters of the hematological profile (p>0.05). However, it also decreased CD4+ and CD4+/CD8+ ratio (p<0.05), besides increasing CD8+ (p<0.05).

**Conclusion:**

Theileriosis does not change the hematological profile of Bali cattle except for the hemoglobin levels. Moreover, it promotes T-cell depletion.

## Introduction

Theileriosis is a tick-borne disease that occurs in tropical areas including Indonesia. It can infect domesticated and wild animals. In cattle, theileriosis is caused by *Theileria parva*, *Theileria* *orientalis*, and *Theileria* *annulata*. Theileriosis causes parasitemia in the host, and the parasite’s life cycle leads to the destruction of lymphocytes and erythrocytes [1]. Moreover, theileriosis causes minimal changes in host antibody synthesis and nutrient distribution. In livestock, most cases of theileriosis result in host growth disorders or death, besides, high economic losses resulting from the production of low-quality products such as meat and milk [2].

Bali cattle are an indigenous beef breed originally from Bali, Indonesia. This breed is well adapted to a high stress production system with high temperature and rainfall, and low feed input [3]. In recent years, Bali cattle distribution has spread to Java, Kalimantan, Sulawesi, and Sumatra. In Indonesia, this breed still represents 27% of the total national cattle population, and there are still some efforts to increase its population [4]. As a local breed, Bali cattle are resistant to many parasitic diseases such as those caused by nematodes [5]; however, no studies have reported its resistance to theileriosis. Resistance profile against hemoprotozoan infections can be detected through hematological analyses. A previous study reported that theileriosis alters the profile of erythrocytes and hematocrits of indigenous cattle in Korea [6]. However, this has not yet been analyzed in Bali cattle, the indigenous cattle in Indonesia.

This study aimed to analyze the hematological profile of Bali cattle from Muara Bulian, Jambi, Indonesia, during natural infection by *Theileria* spp.

## Materials and Methods

### Ethical approval

The animal procedure herein used was approved by the Ethical Clearance Committee, and it was performed following the Guidelines of Animal Use of the Faculty of Animal Husbandry, University of Jambi, Indonesia.

### Sample collection

Blood samples were collected from July to December 2018 from Bali cattle on traditional farms in the subdistrict of Muara Bulian, Batang Hari, Jambi Province, Indonesia. The number of collected samples was calculated using the following formula described by Charan and Biswas [7]:


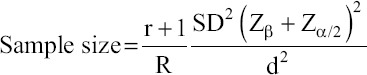


Based on the use of this formula, a total of ninety-four 2-year-old Bali cattle were selected for the collection of blood samples. A cluster random sampling method was performed, i.e., a cattle population from each village was considered as one cluster; Bali cattle individuals were randomly selected from each cluster until the number of 94 individuals was reached. The blood samples were collected from the individuals’ jugular vein (5 ml) using a vacuum blood tube. The collected blood samples were stored in a cooler box and transported to the laboratory for examination.

### Routine blood tests

Routine blood tests were performed using an automated hematology analyzer in the Laboratory of Clinical Pathology, Faculty of Animal Husbandry, University of Jambi, Indonesia. These tests were performed against as the parameters; total erythrocytes, hemoglobin, hematocrit, mean corpuscular volume (MCV), mean corpuscular hemoglobin (MCH), mean corpuscular hemoglobin concentration (MCHC), total leukocytes, lymphocytes, and neutrophils.

### Detection of theileriosis

The detection of theileriosis was performed using blood smear examination. A drop of blood was placed at the end of a glass slide; a second slide at an angle of 40° was used as the spreader, and the blood was smeared from one end of the first slide to the other. The blood smear was aerated until dry and then fixed using methanol. The blood smear was then stained using 10% of Giemsa for 30 min. The stained material was examined under a microscope (1000×).

### Double staining immunohistochemistry (IHC)

Blood samples were transferred to microhematocrit tubes and centrifuged at 10,000 rpm for 5 min. Then, each microhematocrit tube was gently broken, and the buffy coat was placed on a glass slide. The buffy coat was smeared on the glass slide and fixed using methanol. Double staining IHC was performed against CD4+ and CD8+ lymphocytes following the procedure described in Chen *et al*. [8].

All IHC slides were analyzed by a single pathologist. Morphometric analysis was performed by determining the proportion of smear area (%) that expressed CD4+ and CD8+ using the Image J software and by calculating the CD4+/CD8+ ratio. The results were recorded as mean values and standard deviation.

### Statistical analysis

The prevalence of theileriosis was measured as a percentage. The data obtained with the routine hematological tests and IHC were analyzed by performing univariate and bivariate analyses in a statistical software (Statistix version 8.1) provided by Analytical SoftwareInc, Canada.

### Results

A total of 34.04% (32/94) samples were identified as containing theileriosis. Round and oval forms of piroplasms possibly belonging to *T. annulata* were identified in the cytoplasm of erythrocytes. However, the identification as *T. annulata* is not conclusive as no molecular tests were performed.

**Figure-1:**
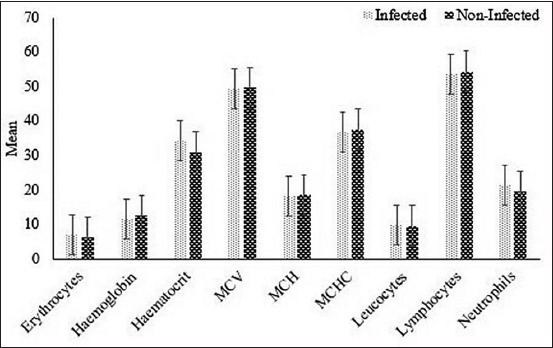
Hematological profile of Bali cattle with and without theileriosis.

The results of the hematology tests are shown in Figure-1, these results were categorized as normal or abnormal depending on the obtained values compared to the study by Suharti *et al*. [9]. A significant correlation (p=0.018) between theileriosis and hemoglobin concentration was found. Nevertheless, the infected cattle displayed no clinical signs. Other values from the hematological tests such as erythrocytes, hematocrits, MCV, MCH, MCHC, lymphocytes, and neutrophils were not correlated with theileriosis in Bali cattle (p>0.05) (Table-1).

**Table 1:** Correlation between Bali cattle hematological profile variables and theileriosis.

Variable	Information	Theileriosis		χ^2^	p-value

		Negative	Positive		
Erythrocytes	Abnormal	37	23	1.360	0.244
	Normal	25	9		
Hemoglobin	Abnormal	21	19	5.616	0.018*
	Normal	41	13		
Hematocrit	Abnormal	45	22	0.151	0.697
	Normal	17	10		
MCV	Abnormal	39	23	0.757	0.384
	Normal	23	9		
MCH	Abnormal	41	22	0.066	0.798
	Normal	21	10		
MCHC	Abnormal	38	17	0.580	0.446
	Normal	24	15		
Leukocytes	Abnormal	38	21	0.170	0.680
	Normal	24	11		
Lymphocytes	Abnormal	17	9	0.005	0.942
	Normal	45	23		
Neutrophil	Abnormal	49	24	0.198	0.657
	Normal	13	8		

* p<0.05 indicates significant differences. MCV=Mean corpuscular volume, MCH=Mean corpuscular hemoglobin, MCHC=Mean corpuscular hemoglobin concentration

IHC showed significant differences (p<0.05) regarding CD4+, CD8+, and the CD4+/CD8+ ratio (Table-2), proving that theileriosis alters the number of circulatory T cells (CD4+ and CD8+) during infection. This effect is probably connected to lymphocyte depletion during the life cycle of *Theileria* spp.

**Table 2:** Immunohistochemistry of CD4+, CD8+, and CD4+/CD8+lymphocytes ratio from blood samples of Bali cattle.

Parameter	Theileriosis (Mean±SD)		p-value

	Negative	Positive	
CD4+(%)	30.866±6.015	18.692±4.081	0.000*
CD8+(%)	18.377±3.138	20.362±3.227	0.005*
CD4+/CD8+	1.721±0.421	0.927±0.189	0.000*

SD=Standard deviation,

* p<0.05 indicates significant differences

## Discussion

Tropical areas present suitable conditions for the expansion and transmission of parasites including hemoprotozoans. Hemoprotozoan parasite infection is caused by tick bites [10]. Several tick species including *Hyalomma* spp., *Rhipicephalus* spp., and *Haemaphysalis* spp. not only transmit hemoprotozoans but they also concomitantly aggravate the infection through hematological profile disturbance [11]. A control strategy must be conducted to prevent the spreading of these parasites. Although a curative strategy has been conducted in Indonesia, it was not effective enough to decrease the prevalence of the theileriosis. Therefore, an epidemiological study must be conducted to provide data on types of parasite infection, parasitemia level, prevalence, and risk factors that influence hemoprotozoan infection. This study showed that the prevalence of theileriosis (34.04%) among the Bali cattle from Muara Bulian is high.

The high prevalence of theileriosis in Muara Bulian and worldwide is suspected to be a result of sociodemographic change (human and animal migration) [12], land diversion [13], and climatic conditions that affect the tick life cycle [14]. These factors increase the resistance of ticks in the environment. Ticks are known to actively search for hosts in warm room temperature (35.0-38.0°C) [15]. This agrees with the results herein found for blood samples collected from July to December, a period of warm room temperature (34.0-37.0°C) in Muara Bulian. The increase of tick population in the environment is followed by the development of infections by hemoprotozoan parasites such as *Theileria* spp. [16]. Theileriosis then alters its host’s hematological profile.

When infecting a host, *Theileria* spp. invades lymphoblasts and becomes a schizont. The schizont then undergoes schizogony and merogony and releases merozoites, which infect other erythrocytes and transform into piroplasms [17]. Through these mechanisms, theileriosis causes severe hematological defects in cattle. Surprisingly, theileriosis did not significantly affect Bali cattle hematological profile, only the animals’ hemoglobin levels. Hemoglobin is the main intracellular constituent of erythrocytes, and its level can be used as an indicator of anemia in bovines [18]. In Bali cattle, *Theileria* spp. causes regenerative anemia, which can be identified based on the depletion of hemoglobin without the depletion of erythrocytes and hematocrits [19]. The fact that theileriosis did not change the hematological profile of Bali cattle is suspected to be due to low degrees of *Theileria* infection.

A more dangerous problem occurs when the immune system fails to destroy both infected cells and circulatory blood parasites. The impairment of T cells, which directly kill and concomitantly induce phagocytosis of parasites, promote severe infection and lead to persistent infection [20]. Both T cells (CD4+ and CD8+) regulate the healing of the host and the decrease of parasite infection through the reduction of host injuries [21]. A previous study reported that CD8+ lymphocytes not only initiate cell destruction but they also have prominent effects that protect the host’s body from future infections [22].

The present study provides information regarding the effects of theileriosis on Bali cattle. Theileriosis does not affect hematological profile except for hemoglobin level; however, it generally promotes CD4+ depletion. The life cycle of *Theileria* spp., which causes lysis of erythrocytes and lymphoblasts, increases circulatory CD8+ to kill infected cells through major histocompatibility complex Class I. Chronic theileriosis is suspected to result in hypercytotoxicity and oxidative stress, which impair the performance of Bali cattle. The impacts of theileriosis can be avoided through the elimination of causative agents and control of vectors. Nevertheless, the infected cattle can be a reservoir of *Theileria* spp. Although this study showed that Bali cattle are resistant to *Theileria* infection, the handling, controlling, and eradication of theileriosis in Bali cattle must be conducted.

## Conclusion

The present study showed and discussed that there is a high prevalence of theileriosis on Bali cattle in the subdistrict of Muara Bulian, Jambi Province, Indonesia. Theileriosis did not affect the hematological profile of Bali cattle except for their hemoglobin levels. However, it significantly affected CD4+, CD8+, and CD4+/CD8+ ratio. Both clinical examination and hematological profile may not be reliable tools for the detection of reservoirs or carrier animals. Thus, the use of more sensitive and specific molecular diagnostic tools is necessary in future studies.

## Authors’ Contributions

NA and MM contributed to experiment design and sample collection. YAP contributed to statistical analysis and data interpretation. NA, MM, and YAP collaborated in writing, revising, and improving the manuscript for publication. All authors read and approved the final version of this manuscript.
